# The effect of the underlying malignancy on short- and medium-term survival of critically ill patients admitted to the intensive care unit: a retrospective analysis based on propensity score matching

**DOI:** 10.1186/s12885-021-08152-5

**Published:** 2021-04-15

**Authors:** Zhen-nan Yuan, Hai-jun Wang, Yong Gao, Shi-ning Qu, Chu-lin Huang, Hao Wang, Hao Zhang, Quan-hui Yang, Xue-zhong Xing

**Affiliations:** Department of Intensive Care Unit, National Cancer Center / National Clinical Research Center for Cancer/Cancer Hospital, Chinese Academy of Medical Sciences & Peking Union Medical College, Beijing, 100021 China

**Keywords:** Malignancy, Intensive care unit, Critical illness, Prognosis

## Abstract

**Background:**

Advances in oncology led to a substantial increase in the number of patients requiring admission to the ICU. It is significant to confirm which cancer critical patients can benefit from the ICU care like noncancer patients.

**Methods:**

An observational retrospective cohort study using intensive care unit (ICU) admissions of Medical Information Mart for Intensive Care III from the Beth Israel Deaconess Medical Center in Boston, MA, USA between 2001 and 2012 was conducted. Propensity score matching was used to reduce the imbalance between two matched cohorts. ICU patients with cancer were compared with those without cancer in terms of patients’ characteristics and survival.

**Results:**

There were 38,508 adult patients admitted to ICUs during the period. The median age was 65 years (IQR, 52–77) and 8308 (21.6%) had an underlying malignancy diagnosis. The noncancer group had a significant survive advantage at the point of 28-day, 90-day, 365-day and 1095-day after ICU admission compared with cancer group (*P* < 0.001 for all) after PSM. Subgroup analysis showed that the diagnosis of malignancy didn’t decrease 28-day and 90-day survive when patients’ age ≥ 65-year, patients in surgical intensive care unit or cardiac surgery recovery unit or traumatic surgical intensive care unit, elective admissions, patients with renal replacement therapy or vasopressor support (*P* > 0.05 for all).

**Conclusions:**

Malignancy is a common diagnosis among ICU patients. Patients without cancer have a survive advantage compared with patients with cancer in the short- and medium-term. However, in selected groups, cancer critical patients can benefit from the ICU care service like noncancer patients in the short-term.

## Background

Historically, the diagnosis of malignancy had been a common reason for rejection of admission to intensive care unit (ICU) because of poor prognosis and high costs [1–3]. Survive of cancer patients has increased over the last 30 years due to a greater awareness of early signs and better therapy [4, 5]. Patients with cancer require ICU admission for severe cancer- or chemo-radiation- or immunotherapy related complications, postoperative care after major surgical resections, and concurrent severe acute illnesses [6, 7]. About five percentage of patients experienced a critical illness resulting in ICU admission within 2 years of cancer diagnosis and around one seventh of patients admitted to general ICUs had a malignancy [6, 8, 9].

The outcome of cancer patients admitted to ICU is strongly dependent on the type of admission. Puxty et al. [10] studied 25,017 surgical admissions to general ICUs in the West of Scotland, and found that ICU and hospital mortality were lower in the group of ICU patients with cancer compared with noncancer patients. Bos et al. [11] reported on the characteristics and outcomes of more than 15,000 cancer patients with an emergency admission to general ICUs and demonstrated that cancer patients have lower hospital survive compared with noncancer patients when admitted because of medical reasons. When physicians decide to admit cancer patients to ICU, it should be remembered that those admitted cancer patients should be likely to benefit from ICU treatments. The challenge facing ICU physicians is to identify which cancer patients are likely to benefit from ICU care like noncancer patients. Most previously published studies didn’t include a comparison group of patients without cancer; thus, it is difficult to determine the effect of cancer within the same ICU or hospital setting [11–13].

Our primary objective was to explore which critical ill patients with cancer can benefit from the ICU care like patients without cancer within the same hospital setting.

## Methods

### Clinical database

The MIMIC-III database is a freely available database comprising more than 40,000 patients in the ICU of the Beth Israel Deaconess Medical Center in Boston, MA, USA between 2001 and 2012. The acquisition of cancer patients is based on the malignant information contained in the admission diagnosis (ICD-9) in the database. Our access to the database was approved after completion of the Collaborative Institutional Training Initiative (CITI program) web-based training course called “Data or Specimens Only research” (Record ID: 36067767).

### Data extraction

Structured Query Language (SQL) with PostgreSQL (version 9.6) was used to extract data from MIMIC-III. Demographics (sex, age, ethnicity), ICU type, admission group (elective and emergency), reasons for ICU admission were extracted for adult patients (≥18 years) admitted to ICU. The severity of illness score was evaluated by the Sequential Organ Failure Assessment (SOFA) score [14], Simplified Acute Physiology Score II (SAPSII) [15], Logistic Organ Dysfunction Score (LODS) [16], Oxford Acute Severity of Illness Score (OASIS) [17] and Acute Physiology Score III (APS III) [18]. For parameters of these five scoring systems, only data within 24 h after ICU admission were extracted and missing components for calculation were treated as normal (usually zero). Comorbidities were evaluated using the Elixhauser comorbidity system which scores a series of comorbid diseases and conditions according to the severity of organ decompensation and prognostic impact [19], the higher the score, the worse the patient’s health. Other extracted data included mechanical ventilation (MV), vasopressor usage, renal replacement therapy (RRT), sepsis, hospital infection. The endpoints of our study were 28-day, 90-day, 365-day and 1095-day survive after ICU admission.

### Population selection criteria

Inclusion criteria: (1) Enrolled cancer subjects were patients with diagnosis of malignancy according to International Classification of Diseases, Ninth Revision code (ICD-9). (2) Patients whose age < 18 were excluded. (3) The first ICU stay of the first hospitalization.

**Fig. 1:**
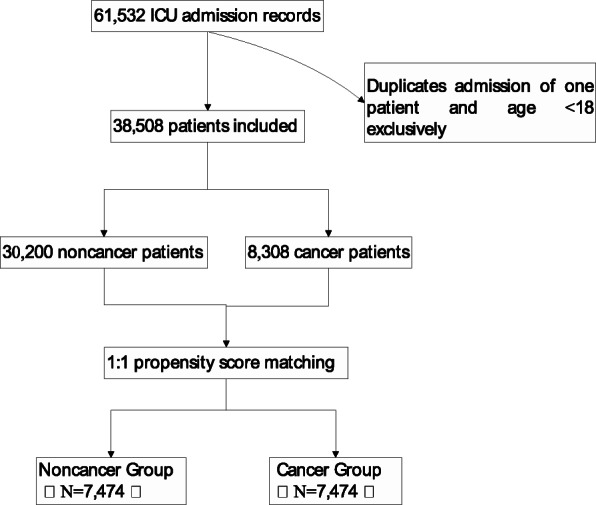
Flow plot of data extraction and filtration from the MIMIC-III database

Suspected infection [20] was based on the International Classification of Diseases, Ninth Revision, Clinical Modification codes (ICD-9-CM). The diagnosis of sepsis was identified using the third international consensus definitions for sepsis and Septic Shock (Sepsis-3) [21]. The screening process of enrolled patients is shown in Fig. 1.

### Statistical analysis

Categorical variables were described as the number and percentage, and their differences among groups were compared using Chi-squared test. Noncontinuous variables and continuous variables that didn’t follow normal distribution were descried as median and interquartile range (IQR), and were analyzed with non-parametric methods (Mann-Whitney-Wilcoxon for two groups, Kruskal-Wallis for multi-groups). Continuous variables that followed normal distribution were expressed as mean and standard deviations, and t-test (two groups) or Analysis of Variance (ANOVA) (multiple groups) was used for these variables. Kaplan-Meier curves were used to calculate the survive rate and the log-rank test was used for comparisons among the overall population and subgroups. We performed this study via available MIMIC database based on propensity score matching (PSM). The use of PSM aimed to reduce the imbalance between two matched cohorts. The factors included in the PSM were as follows: sex, ethnicity, ICU type, admission group, admission reasons, SOFA, SAPS II, APS III, LODS, OASIS, Elixhause comorbidity, RRT on first day, MV on first day, vasopressor administration on first day, sepsis, infection.

Standardized difference (SDD) before and after matching were plotted to show the effect of matching. In the cohort of propensity score-matched subjects, the SDD of all covariates between cancer group and noncancer group were < 10%, which suggested that the PSM appropriately adjusted for the initial selection bias. The bias in subgroups after successful PSM could also be considered as balanced [22]. A *P* value < 0.05 was considered statistically significant. Statistical analyses were performed using Stata, version 14.0 (Stata Corp).

## Results

### Characteristics of the patients with cancer and without cancer

**Fig. 2:**
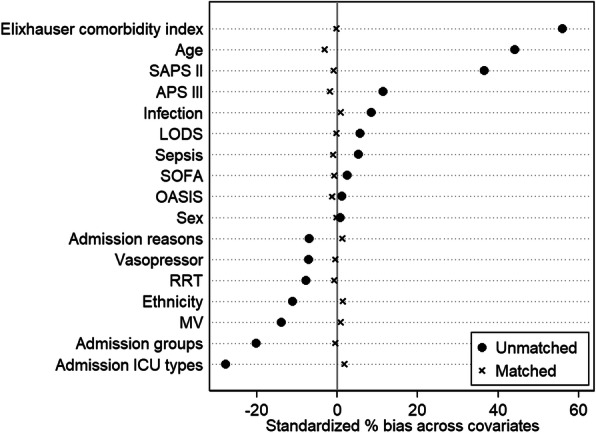
Balance of covariates between critical care patients with and without cancer and before and after propensity score matching. (Abbreviations: Sequential Organ Failure Assessment, SOFA; Simplified Acute Physiology Score II, SAPSII; Logistic Organ Dysfunction Score, LODS; Oxford Acute Severity of Illness Score, OASIS; Acute Physiology Score, APSIII; intensive care unit, ICU; mechanical ventilation, MV; renal replacement therapy, RRT)

During this study period, there were 38,508 adult patients admitted to ICUs in the Beth Israel Deaconess Medical Center in Boston, MA, USA between 2001 and 2012, of whom 8308 (21.6%) had an underlying malignancy diagnosis. The median age was 65 years (IQR, 52–77) and 16,715 (43.4%) patients were female. Table 1 describes patients’ characteristics for admissions to ICU with and without a diagnosis of malignancy before and after PSM. ICU patients with cancer were older than noncancer patients with median (IQR) age 70 (60–79) VS 63 (50–77) years (*P* < 0.001). The percentage of patients without cancer admitted to ICU as an emergency was 85.9% (25,993 of 30,200 patients) in contrast to 78.1% (6485 of 8308 patients) of the population with cancer. Compared with noncancer patients, patients with cancer were more likely to be admitted by surgical intensive care unit (SICU) (21.5% VS 15.2%) and medical intensive care unit (MICU) (42.0% VS 33.6%). The percentage of the noncancer patients in coronary care unit (CCU), cardiac surgery recovery unit (CSRU) and traumatic surgical intensive care unit (TSICU) were more than that of cancer patients. The admission reasons were similar between the cancer and noncancer groups. Cancer patients tended to have higher critical illness score compared with noncancer patients, SAPSII [39(26–46) VS 31(23–41), *P* < 0.001], APSIII [40(30–53) VS 37(27–51), *P* < 0.001]. The Elixhauser comorbidity index score of cancer group was also significantly higher than noncancer group [9(2–17) VS 3(0–9), *P* < 0.001]. Respiratory support was the most common organ support for both the cancer and noncancer groups at 40.3% (3351 of 8308 patients) and 47.2% (14,262 of 30,200 patients), respectively. Cardiovascular support was provided to 27.0% of the cancer group (2240 of 8308 patients) and 30.2% of the noncancer group (9114 of 30,200 patients). Renal replacement therapy was not frequently used in either group, but those patients in the cancer group had a lower percentage of RRT [147 of 8308 patients (1.8%)] compared with the noncancer group [889 of 30,200 patients (2.9%), *P* < 0.001]. Patients with cancer were more likely to have a higher frequency of hospital infection (42.7% VS 38.6%, *P* < 0.001) and sepsis (7.8% VS 6.5%, *P* < 0.001). After the PSM using 1:1, the covariates of the cancer group and noncancer group were balanced with a standard difference less than 5% (Fig. 2).

**Table 1:** Comparison of variables between Intensive care patients with cancer and without cancer before and after propensity score matched analysis

Variable	ALL	Patients before PSM				Patients after PSM			
	*N* = 38,508	Noncancer(*N* = 30,200)	Cancer (*N* = 8308)	*P* Value	SDD	Noncancer *N* = 7474	Cancer *N* = 7474	*P* Value	SDD
Age, median (IQR), Y	65 (52–77)	63 (50–77)	70 (60–79)	< 0.001	44.2	72 (59–81)	69 (59–79)	1.000	−3.3
Sex, No. (%)				0.545	0.8			0.921	0
Female	16,715 (43.4)	13,133 (43.5)	3582 (43.1)			3276 (43.8)	3282 (43.9)		
Male	21,783 (56.6)	17,067 (56.5)	4726 (56.8)			4198 (56.2)	4192 (56.1)		
Ethnicity, No. (%)				< 0.001	11.1			0.829	1.6
Black	2949 (7.7)	2404 (8.0)	545 (6.6)			531 (7.1)	505 (6.8)		
Asian	911 (2.4)	667 (2.2)	244 (2.9)			195 (2.6)	207 (2.8)		
White	27,468 (71.3)	21,035 (69.7)	6433 (77.4)			5749 (76.9)	5735 (76.7)		
Hispanic	1254 (3.3)	1095 (3.6)	159 (1.9)			154 (2.1)	154 (2.1)		
Other	5926 (15.4)	4999 (16.6)	927 (11.2)			845 (11.3)	873 (11.7)		
ICU type, No. (%)				< 0.001	27.7			0.717	1.7
SICU	6366 (16.5)	4578 (15.2)	1788 (21.5)			1528 (20.4)	1499 (20.1)		
MICU	13,634 (35.4)	10,148 (33.6)	3486 (42.0)			3090 (41.3)	3064 (41.0)		
CCU	5673 (14.7)	4728 (16.7)	945 (11.4)			906 (12.1)	920 (12.3)		
CSRU	7606 (19.8)	6378 (21.1)	1228 (14.8)			1215 (16.3)	1207 (16.2)		
TSICU	5229 (13.6)	4368 (14.5)	861 (10.4)			735 (9.8)	784 (10.5)		
Admission group, No. (%)				< 0.001	−20.2			0.764	−0.4
Elective	6090 (15.8)	4267 (14.1)	1823 (21.9)			1465 (19.6)	1467 (19.6)		
Emergency	32,418 (84.2)	25,993 (85.9)	6485 (78.1)			6009 (80.4)	6007 (80.4)		
Admission reasons, No. (%)				< 0.001	−7.0			0.860	1.4
Cardiovascular	17,528 (45.5)	13,726 (45.5)	3802 (45.8)			3419 (45.8)	3416 (45.7)		
Liver	1312 (3.4)	903 (3.0)	409 (4.9)			330 (4.4)	328 (4.4)		
Mental	4118 (10.7)	3300 (10.9)	818 (9.9)			783 (10.5)	771 (10.3)		
Renal	4753 (12.3)	3686 (12.2)	1067 (12.8)			1011 (13.6)	970 (13.0)		
Respiratory	6905 (18.0)	5478 (18.2)	1427 (17.2)			1239 (16.6)	1288 (17.2)		
Coagulation	1671 (4.3)	1257 (4.2)	414 (5.0)			366 (4.9)	356 (4.8)		
Other	2221 (5.8)	1850 (6.1)	371 (4.5)			326 (4.4)	345 (4.6)		
SOFA, median (IQR)	3 (2–5)	3 (2–5)	3 (2–5)	0.001	2.5	3 (2–6)	3 (2–6)	0.622	−0.9
SAPS II, median (IQR)	24 (33–92)	31 (23–41)	39 (26–46)	< 0.001	36.6	36 (28–46)	36 (28–44)	0.490	−1.4
APS III, median (IQR)	38 (25–51)	37 (27–51)	40 (30–53)	< 0.001	11.4	40 (29–54)	39 (30–52)	0.462	−2.0
LODS, median (IQR)	3 (2–5)	3 (2–5)	3 (2–5)	< 0.001	5.7	4 (2–5)	3 (2–5)	1.00	−0.5
OASIS, median (IQR)	30 (25–37)	30 (25–37)	30 (25–37)	0.293	1.2	31 (25–37)	31 (25–37)	0.09	−1.4
Elixhause comorbidity, median (IQR)	4 (0–12)	3 (0–9)	9 (2–17)	< 0.001	56.0	9 (0–16)	8 (0–15)	0.933	−0.4
RRT on first day, No. (%)	1036 (2.7)	889 (2.9)	147 (1.8)	< 0.001	−7.7	154 (2.1)	145 (1.9)	0.599	−0.7
MV on first day, No. (%)	17,613 (45.7)	14,262 (47.2)	3351 (40.3)	< 0.001	−13.9	3051 (40.8)	3084 (41.3)	0.583	1.1
Vasopressor administration on first day, No. (%)	11,354 (29.5)	9114 (30.2)	2240 (27.0)	< 0.001	−7.1	2094 (28.0)	2076 (27.8)	0.743	−1.1
Sepsis, No. (%)	2601 (6.8)	1951 (6.5)	650 (7.8)	< 0.001	5.3	603 (8.1)	783 (7.8)	0.545	−1.4
Infection, No. (%)	15,192 (39.5)	11,641 (38.6)	3551 (42.7)	< 0.001	8.5	3164 (42.3)	2196 (42.8)	0.597	1.1

Abbreviations: *ICU* Intensive care unit; *SDD* The standardized differences; *PSM* Propensity score matching; *IQR* 25–75% Interquartile range; *SOFA* Sequential Organ Failure Assessment; *SAPSII* Simplified Acute Physiology Score II; *LODS* Logistic Organ Dysfunction Score; *OASIS* Oxford Acute Severity of Illness Score; *APSIII* Acute Physiology Score; *SICU* Surgical intensive care unit; *MICU* Medical intensive care unit; *CCU* Coronary care unit; *CSRU* Cardiac surgery recovery unit; *TSICU* Traumatic surgical intensive care unit; *MV* Mechanical ventilation; *RRT* Renal replacement therapy

### Survival outcomes of enrolled patients before and after PSM analysis (Table 2)

**Fig. 3:**
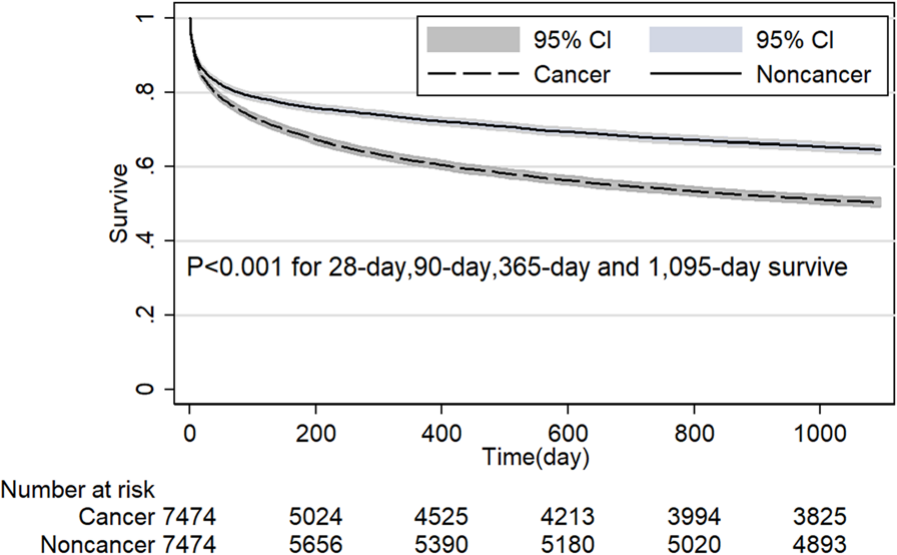
Comparison of Kaplan-Meier survival curves between critical care subjects with and without cancer in overall population after propensity score matching

Before PSM analysis, 28-day, 90-day, 365-day and 1095-day survive rate of noncancer group were 88.0, 84.3, 79.1 and 72.6%, respectively, which were higher than the cancer group with 81.3, 72.9, 59.3 and 48.3%, respectively. After PSM analysis, the noncancer group still had a significant survive advantage at the point of 28-day, 90-day, 365-day and 1095-day after admission compared with cancer group (*P* < 0.001 for all) in the overall population (Fig. 3). Cancer patients had a similar length of ICU stay and slightly longer hospital stay (7.3-day VS 6.8-day, *P < 0.001*) compared with noncancer patients.

**Table 2:** Outcomes of patients with cancer and without cancer before and after propensity score matched analysis

Outcome	ALL	Patients before PSM			Patients after PSM		
	*n* = 38,508	Noncancer *N* = 30,200	Cancer *N* = 8308	*P* Value	Noncancer *N* = 7474	Cancer *N* = 7474	*P* Value
LOS of ICU, median (IQR), d	2.1 (1.2–4.1)	2.1 (1.2–4.1)	2.1 (1.2–3.9)	< 0.001	2.1 (1.2–4.2)	2.1 (1.2–3.9)	< 0.001
LOS of Hospital, median (IQR), d	6.9 (4.0–11.9)	6.8 (4.0–11.8)	7.3 (4.4–12.2)	< 0.001	7.1 (4.2–12.6)	7.2 (4.3–11.9)	0.774
28-day survive, % (95%CI)	86.6 (86.2–86.9)	88.0 (87.6–88.4)	81.3 (80.5–82.1)	< 0.001	84.9 (84.0–85.7)	82.3 (81.4–83.1)	< 0.001
90-day survive, % (95%CI)	81.8 (81.4–82.2)	84.3 (83.9–84.7)	72.9 (72.0–73.9)	< 0.001	79.2 (78.3–80.1)	74.3 (73.3–75.3)	< 0.001
365-day survive, % (95%CI)	74.9 (74.4–75.3)	79.1 (78.7–79.6)	59.3 (58.2–60.3)	< 0.001	72.7 (71.7–73.7)	61.4 (60.3–62.5)	< 0.001
1095-day survive, % (95%CI)	67.3 (66.9–67.8)	72.6 (72.1–73.1)	48.3 (47.3–49.4)	< 0.001	64.6 (63.5–65.7)	50.4 (49.3–51.6)	< 0.001

Abbreviations: *LOS* Length of stay; *ICU* Intensive care unit; *PSM* Propensity score matching; *IQR* 25–75% Interquartile range

### Subgroups analysis of short and medium-term survival of critically ill patients between with cancer and without cancer after PSM (Table 3)

**Fig. 4:**
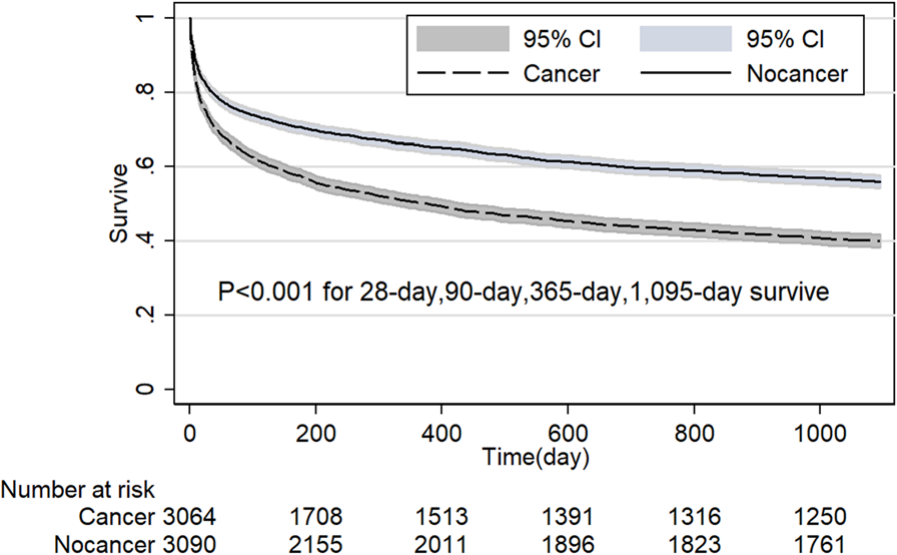
Comparison of Kaplan-Meier survival curves between critical care subjects with and without cancer in MICU after propensity score matching. (Medical intensive care unit, MICU)

**Fig. 5:**
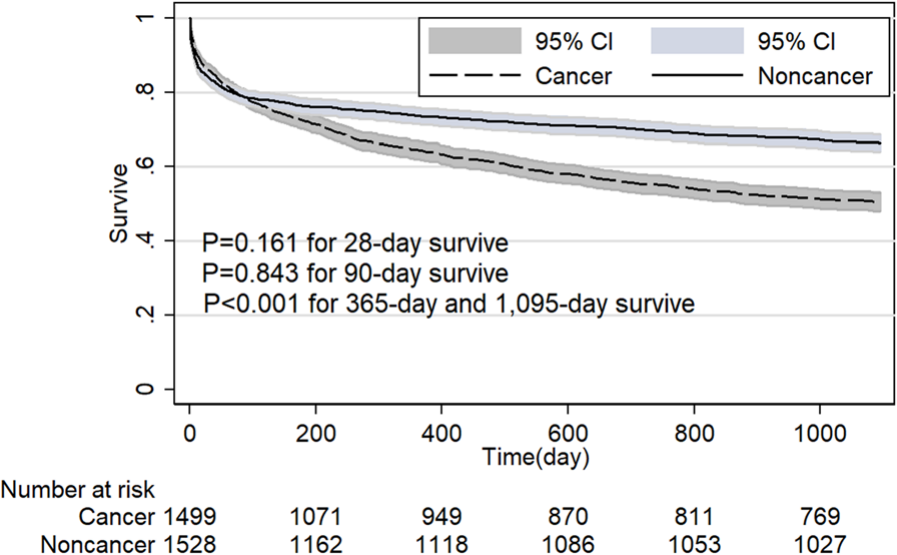
Comparison of Kaplan-Meier survival curves between critical care subjects with and without cancer in SICU after propensity score matching. (Surgical intensive care unit, SICU)

**Fig. 6:**
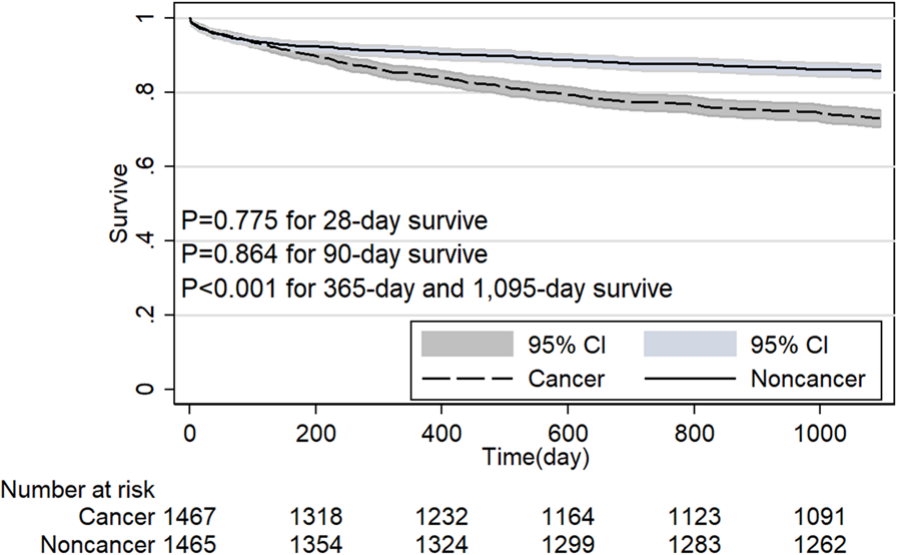
Comparison of Kaplan-Meier survival curves between critical care subjects with and without cancer in elective admissions after propensity score matching

**Fig. 7:**
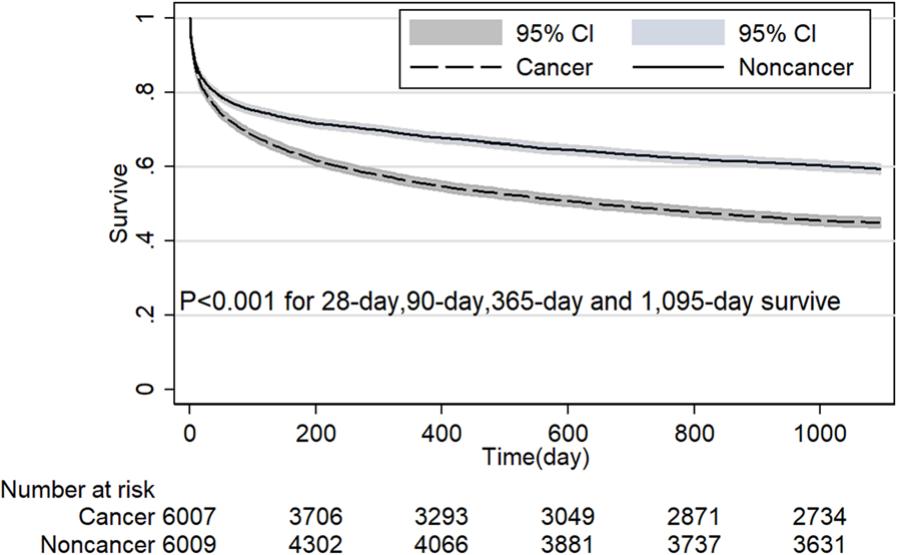
Comparison of Kaplan-Meier survival curves between critical care subjects with and without cancer in emergency admissions after propensity score matching

The noncancer group had a significant survive advantage than cancer group in MICU (*P* < 0.001, Fig. 4). However, when patients admitted by SICU, the survive advantage disappeared at the point of 28-day and 90-day (*P* > 0.05 for both, Fig. 5). Table 3 shows 28-day and 90-day survive in patients with and without cancer stratified based on distinct patient subgroups. Compared with noncancer group, 28-day and 90-day survive rate was lower in the cancer group when patients’ age < 65-year, black, in MICU, emergency admission (Fig. 6), admission because of cardiovascular or respiratory or coagulation dysfunction, Elixhauser comorbidity index score ≥ 4, and patients without RRT or vasopressor support (*P* < 0.05 for all). 28-day and 90-day survive rate showed no difference between cancer and noncancer group when patients’ age was older than 65-year, patients in SICU or CSRU or TSICU, elective admission (Fig. 7), patients with RRT or vasopressor support (*P* > 0.05 for all). Although when patients were Asian or White or Hispanic, admission because of liver or mental or renal disorder, Elixhauser comorbidity index score < 4, patients with cancer showed no different 28-day survive compared with noncancer patients (*P* > 0.05 for all), the survive advantage appeared at 90-day survive for noncancer patients (*P* < 0.05 for all). However, for patients in CCU, cancer group has higher 28-day survive rate than the noncancer group (*P* = 0.022) and the survive advantage disappeared at the point of 90-day (*P* = 0.409).

**Table 3:** 28-day and 90-day Survive in Patients With and Without Cancer by Admission Features after propensity score matching

Variable	28-day survive, % (95% CI)			90-day survive, % (95% CI)		
	Noncancer	Cancer	*P* Value	Noncancer	Cancer	*P* Value
Age						
< 65	92.2 (91.1–93.1)	84.3 (82.9–85.6)	< 0.001	90.2 (88.9–91.2)	76.7 (75.1–78.3)	< 0.001
≥ 65	80.9 (79.7–82.0)	81.1 (79.9–82.2)	0.697	73.3 (72.0–74.5)	72.9 (71.6–74.1)	0.791
Sex						
Female	83.6 (82.2–84.8)	80.2 (78.8–81.5)	0.001	78.2 (76.8–79.6)	72.3 (70.8–73.8)	< 0.001
Male	85.9 (84.8–86.9)	83.9 (82.8–85.0)	0.012	80.0 (78.8–81.2)	75.9 (74.5–77.1)	< 0.001
Ethnicity						
Black	90.2 (87.4–92.5)	80.4 (76.7–83.6)	< 0.001	84.0 (80.6–86.9)	69.9 (65.7–73.7)	< 0.001
Asian	85.2 (79.3–89.4)	80.6 (74.6–85.4)	0.215	80.0 (73.7–85.0)	71.0 (64.3–76.7)	0.036
White	84.9 (83.9–85.8)	83.7 (82.7–84.6)	0.111	79.4 (78.3–80.4)	76.1 (75.0–77.2)	< 0.001
Hispanic	90.3 (84.4–94.0)	86.4 (79.9–90.9)	0.293	87.7 (81.3–92.0)	74.7 (67.0–80.8)	0.005
Other	80.2 (77.4–82.8)	73.9 (70.8–76.7)	0.002	73.5 (70.4–76.3)	66.0 (62.7–69.0)	0.001
ICU type						
SICU	84.4 (82.5–86.2)	86.1 (84.3–87.8)	0.161	78.6 (76.5–80.6)	78.6 (76.4–80.6)	0.843
MICU	81.6 (80.1–82.9)	73.8 (72.2–75.3)	< 0.001	74.3 (72.8–75.8)	63.5 (61.8–65.2)	< 0.001
CCU	81.9 (79.2–84.3)	85.7 (83.2–87.8)	0.022	77.4 (74.5–80.0)	78.5 (75.7–81.0)	0.409
CSRU	95.5 (94.1–96.5)	94.4 (92.9–95.5)	0.218	93.2 (91.6–94.5)	91.2 (89.5–92.7)	0.076
TSICU	85.7 (83.0–88.1)	85.6 (82.9–87.9)	0.978	80.3 (77.2–83.0)	77.3 (74.2–80.1)	0.216
Admission group						
Elective	96.8 (95.7–97.5)	96.5 (95.5–97.4)	0.774	94.1 (92.7–95.2)	94.2 (92.9–95.3)	0.864
Emergency	81.8 (80.8–82.7)	78.7 (77.6–79.7)	< 0.001	75.6 (74.5–76.7)	69.5 (68.3–70.6)	< 0.001
Admission reasons						
Cardiovascular	86.2 (85.0–87.3)	83.4 (82.1–84.6)	0.003	82.0 (80.7–83.2)	76.5 (75.0–77.9)	< 0.001
Liver	83.0 (78.5–86.7)	78.6 (73.8–82.7)	0.122	75.8 (70.8–80.0)	62.2 (56.7–67.2)	< 0.001
Mental	81.1 (78.2–83.7)	77.7 (74.6–80.5)	0.090	74.0 (70.7–76.9)	68.7 (65.3–71.8)	0.020
Renal	76.6 (73.8–79.1)	78.3 (75.5–80.7)	0.374	67.1 (64.1–69.9)	69.0 (66.0–71.8)	0.336
Respiratory	87.4 (85.4–89.1)	84.4 (82.3–86.3)	0.003	82.2 (80.0–84.3)	78.7 (76.4–80.9)	0.026
Coagulation	87.7()83.9–90.7	78.9 (74.2–82.9)	0.001	79.5 (75.0–83.0)	68.3 (63.2–72.8)	0.001
Other	94.8 (91.8–96.7)	91.6 (88.1–94.1)	0.116	92.3 (88.9–94.8)	81.7 (77.2–85.4)	< 0.001
SOFA,						
<3	93.0 (91.9–93.8)	90.4 (89.2–91.3)	0.001	89.6 (88.4–90.7)	82.4 (81.0–83.8)	0.003
≥ 3	80.1 (78.9–81.2)	77.4 (76.2–78.6)	0.002	73.1 (71.8–74.4)	69.5 (68.1–70.8)	< 0.001
SAPS II						
<24	98.1 (97.1–98.7)	96.7 (95.4–97.7)	0.044	97.3 (96.2–98.1)	93.2 (91.5–94.6)	< 0.001
≥ 24	82.4 (81.5–83.4)	80.1 (79.1–81.1)	0.001	75.9 (74.8–76.9)	71.5 (70.4–72.6)	< 0.001
APS III						
<38	94.5 (93.6–95.2)	92.8 (91.9–93.6)	0.005	91.6 (90.6–92.5)	86.5 (85.3–87.6)	< 0.001
≥ 38	77.1 (75.8–78.3)	73.6 (72.3–75.0)	0.001	69.2 (67.8–79.6)	64.3 (62.8–65.7)	< 0.001
LODS						
<3	93.9 (92.8–94.7)	90.9 (89.8–92.0)	< 0.001	90.8 (89.6–91.8)	83.5 (82.0–84.8)	< 0.001
≥ 3	80.2 (79.0–81.3)	77.8 (76.6–78.9)	0.006	73.2 (72.0–74.5)	69.6 (68.3–70.8)	< 0.001
OASIS						
<30	95.6 (94.8–96.3)	92.9 (91.9–93.7)	< 0.001	92.0 (91.0–92.9)	86.0 (84.8–87.1)	< 0.001
≥ 30	76.4 (75.1–77.6)	73.7 (72.3–75.0)	0.007	69.1 (67.7–70.5)	64.8 (63.3–66.2)	< 0.001
Elixhause comorbidity						
<4	92.6 (91.6–93.6)	91.7 (90.5–92.7)	0.232	90.8 (89.7–91.9)	86.7 (85.3–88.0)	< 0.001
≥ 4	80.8 (79.7–81.9)	77.6 (76.4–78.7)	< 0.001	73.2 (71.9–74.4)	68.1 (66.7–69.3)	< 0.001
RRT						
Yes	69.5 (61.3–76.3)	71.0 (62.9–77.7)	0.951	61.7 (53.5–68.8)	63.5 (55.1–70.7)	0.930
No	85.2 (84.3–86.0)	82.5 (81.6–83.4)	< 0.001	79.0 (78.7–80.5)	74.5 (73.5–75.5)	< 0.001
MV						
Yes	81.1 (79.7–82.5)	78.5 (77.0–79.1)	0.011	76.3 (74.8–77.8)	72.2 (70.5–73.7)	< 0.001
No	87.4 (86.1–88.4)	84.8 (83.9–86.0)	0.001	81.2 (80.0–82.3)	75.8 (74.5–77.1)	< 0.001
Vasopressor						
Yes	79.1 (77.3–80.8)	77.5 (75.7–79.2)	0.254	74.0 (72.0–75.8)	72.3 (70.3–74.2)	0.249
No	87.1 (86.2–88.0)	84,1 (83.1–85.1)	< 0.001	81.3 (80.2–82.3)	75.1 (73.9–76.2)	< 0.001
Sepsis						
Yes	64.0 (60.0–67.7)	51.3 (47.2–55.3)	< 0.001	52.4 (48.3–56.3)	41.7 (37.7–45.7)	< 0.001
No	86.7 (85.9–87.5)	84.9 (84.0–85.7)	0.005	81.6 (80.6–82.5)	77.1 (76.1–78.1)	< 0.001
Infection						
Yes	78.9 (77.5–80.3)	75.2 (73.6–76.6)	< 0.001	70.0 (68.3–71.5)	64.8 (63.1–66.4)	< 0.001
No	89.2 (88.2–90.1)	87.6 (88.6)	0.027	86.0 (84.9–87.0)	81.4 (80.2–82.6)	< 0.001

Abbreviations: *SOFA* Sequential Organ Failure Assessment; *SAPSII* Simplified Acute Physiology Score II; *LODS* Logistic Organ Dysfunction Score; *OASIS* Oxford Acute Severity of Illness Score; *APSIII* Acute Physiology Score; *SICU* Surgical intensive care unit; *MICU* Medical intensive care unit; *CCU* Coronary care unit; *CSRU* Cardiac surgery recovery unit; *TSICU* Traumatic surgical intensive care unit; *MV* Mechanical ventilation; *RRT* Renal replacement therapy

## Discussion

The interesting point of our research was the inclusion of consecutive admissions of cancer and noncancer patients during the same period. In the crude model, the higher survive rate was found in ICU patients with noncancer than those with cancer in the overall population at the point of 28-day, 90-day, 365-day and 1095-day. Using a one-to-one PSM analysis to address selection bias, we found that the difference was narrowed though noncancer patients still had a slight survival advantage. In this study, we accepted five criticality scores to evaluate the illness of patients. The criticality scores of cancer patients were higher than those of noncancer patients. No matter in the group with high critical illness score or the group with low critical illness score, the 28-day and 90-day survive of cancer patients was significantly worse than that of noncancer patients (*P* < 0.05 for both). ICU patients with cancer tended to be older than patients without cancer and cancer patients usually had a worse outcome when patients’ age > 65. However, the poor outcome wasn’t associated with the diagnosis of malignancy.

To further subgroup analysis of 28-day and 90-day survive associated with cancer diagnosis, we found that the diagnosis of the malignancy didn’t decrease the 28-day and 90-day survival rates in selected groups. When patients were admitted by MICU, 28-day and 90-day survive rate was higher in noncancer patients than cancer patients. However, cancer patients who were admitted by SICU had similar outcomes compared with those of noncancer patients. The finding is consistent with those of previous studies [10, 11]. For cancer patients admitted to MICU, the cancer stage was usually late [13] and the cancer related therapy may lead to immunosuppressive status [23], so the early prognosis was usually poor. However, for patients in SICU, the cancer stage was usually early [13] and the tumor resection have reduced most of the tumor load, so the diagnosis of malignancy had few effects on their short-term prognosis. So we can concluded that short-term survive rates especially differed between cancer and non-cancer patients admitted to ICU because of medical reasons, whereas the difference between cancer and non-cancer patients was not existing in SICU.

Emergency or elective admissions often affect the prognosis of patients in the ICU. In the present study, when patients were admitted as elective admission, it showed no difference of survive rate at the point of 28-day and 90-day between cancer and noncancer patients though the difference appeared at the point of 365-day. When patients were admitted as emergency, the noncancer patients had a survive advantage within 1095 days after ICU admission. Bos et al. [11] also reported the similar outcome. They demonstrated that emergency ICU admission was associated with a survive advantage in patients with noncancer. It was due to a higher incidence of acute comorbidity and a greater severity of illness on admission in the cancer group [11].

In the present study, patients with cancer had a greater need for intensive support (eg, MV, vasopressor administration, and RRT), which was consistent with previous literature [24]. In patients with MV support, we found that noncancer patients had survive advantage at the point of 28-day and 90-day after ICU admission. Hsiue et al. [25] compared 518 patients with solid cancers with 1362 non-cancer patients receiving MV admitted to ICU between 2012 and 2014. They found that the 28-day and 90-day mortality rates were higher in cancer patients than in noncancer patients (45.2% vs. 29.4, and 65.6% vs. 37.7%, respectively, both *P* < 0.001). It remains controversial to start RRT for acute renal failure in critically ill patients with cancer because of the poor outcome and high costs [26–31]. However, the controversy lacked large sample study in previous literature. In our study, for patients in need of RRT, the diagnosis of malignancy didn’t decrease their 28-day and 90-day survive in this study. Benoit et al. [3] and Maccariello et al. [32] also demonstrated that though the early prognosis of cancer patients with acute renal failure in need of RRT was poor, the malignancy itself was not an independent risk factor that affected the prognosis. Cancer patients with shock admitted to ICU usually have a poor prognosis [33]. Previous study reported the in-hospital mortality rate of cancer patients with sepsis shock seemed to be higher than that of noncancer patients [34–36]. However, they did not include a control group of septic shock patients without cancer. In the present study, when cancer patients with RRT or vasopressor support were admitted to ICU, they had worse short-term outcome, but the poor outcome was not caused by the diagnosis of malignancy.

It has found chronic disease burden to be significantly associated with short-term prognosis [37, 38]. We accepted a single numeric score based on the Elixhauser comorbidity index to describe the chronic health status of patients [19]. Cancer patients seems to be with more chronic comorbidities than noncancer patients. In this study, for those patients with poor chronic health or limited functional status, the diagnosis of malignancy had been demonstrated to be associated with poorer 28-day survival. While for patients with better health status (Elixhauser comorbidity score < 4), the survive difference disappeared at the point of 28-day though the noncancer group still had a survive advantage at the point of 90-day. So the diagnosis of malignancy has a more effect on patients with more comorbidities.

Patients with cancer diagnosis were more likely to have infections or sepsis compared with those noncancer patients in this study. It had been reported that about 17% associated with sepsis among cancer patients in MICU [39]. Sepsis is one of the main causes of ICU admission for cancer patients [7] and is an important cause of hospital mortality and morbidity [9]. Treatment of cancer has contributed to a growing number of immunocompromised patients with an increased incidence of hospital infections; immunosuppression can result in a greater use of antibiotics and more infections associated with multi-resistant microorganisms [23]. In our study, cancer patients with hospital infection or sepsis had worse prognosis than noncancer patients with hospital infection or sepsis. Therefore, for patients with malignancy, we should pay attention to the status of potential infections.

The strength of the present study includes the low confounding bias due to equivalence of covariates between the two study groups in the propensity score-matched cohort. The propensity score method creates a model that reflects the effects of risk factors on the exposure [22]. This study also included a comparison group of patients without cancer to determine effect of cancer within the same hospital setting. Our study also had shortcomings, firstly, the cancer stage is not available, which may be a factor that affects the early prognosis of the patients. Secondly, the time of diagnosis of malignancy was not consistent, which may be more than 2 years earlier before their ICU admission. Those patients with more than 5-year tumor free survive can be considered as cured completely.

## Conclusions

Advances in oncology led to a substantial increase in the number of patients requiring admission to the ICU. In the overall population, noncancer patients had a survive advantage of short- and medium-term. When patients’ age ≥ 65 years, patients in SICU or CSRU or TSICU, elective admission, patients with RRT or vasopressor support, the diagnosis of malignancy didn’t decrease their short-term survive rate and cancer patients can benefit from ICU intensive care like noncancer patients. These findings can help us make cancer-related prognosis judgments and make corresponding clinical decisions.

## Data Availability

The datasets used and/or analyzed during the current study are available from the corresponding author (Xue-zhong Xing, e-mail: xueyujuanyanzi@163.com) on reasonable request.

## Abbreviations

ICU: Intensive care unit
SDD: The standardized differences
PSM: Propensity score matching
IQR: 25–75% Interquartile range
SOFA: Sequential organ failure assessment
SAPSII: Simplified acute physiology score II
LODS: Logistic organ dysfunction score
OASIS: Oxford acute severity of illness score
APSIII: Acute physiology score
SICU: Surgical intensive care unit
MICU: Medical intensive care unit
CCU: Coronary care unit
CSRU: Cardiac surgery recovery unit
TSICU: Traumatic surgical intensive care unit
MV: Mechanical ventilation
RRT: Renal replacement therapy
